# Trends of COVID-19 incidence in Manitoba and public health measures: March 2020 to February 2022

**DOI:** 10.1186/s13104-022-06049-5

**Published:** 2022-05-10

**Authors:** Laila Aboulatta, Kaarina Kowalec, Joseph Delaney, Silvia Alessi-Severini, Christine Leong, Jamie Falk, Sherif Eltonsy

**Affiliations:** 1 College of Pharmacy, Rady Faculty of Health Sciences, University of Manitoba, 750 McDermot Avenue, Winnipeg, MB R3E 0T5 Canada; 2 Department of Medical Epidemiology and Biostatistics, Karolinska Institutet, Stockholm, Sweden; 3 Department of Epidemiology, University of Washington, Seattle, USA; 4 Manitoba Center for Health Policy, Winnipeg, MB Canada; 5 Deptarment of Psychiatry, College of Medicine, Rady Faculty of Health Sciences, University of Manitoba, Winnipeg, Canada; 6 The Children’s Hospital Research Institute of Manitoba, Winnipeg, Canada

**Keywords:** COVID-19, Mitigation measures, Masks, Physical distancing, Incidence

## Abstract

**Objectives:**

The increasing spread of severe acute respiratory syndrome coronavirus-2 has prompted Canada to take unprecedented measures. The objective of this study was to examine the impact of the implemented public health measures on the incidence of COVID-19 in Manitoba.

**Results:**

Using the COVID-19 dataset, we examined the temporal trends of daily reported COVID-19 cases and the coinciding public health measures implemented from March 12, 2020 to February 28, 2022. We calculated the 7-day moving average and crude COVID-19 infection rate/100,000 Manitobans. Due to the restrictions applied, the infection rate decreased from 2.4 (April 1) to 0.07 infections (May 1, 2020). Between May 4 and July 17, 2020, the reported cases stabilized, and some restrictions were lifted. However, in November, the cases peaked with infection rate of 29. Additional restrictions were implemented, and the rate dropped to 3.6 infections on March 31, 2021. As of August 2021, 62.8% of eligible Manitobans received two vaccine doses. The infection rate increased to 128.3 infections on December 31, 2021 and mitigation measures were implemented. This study describes how physical distancing in conjunction with other containment measures can reduce the COVID-19 burden. Future studies into the extent of the implementation of the restrictions are necessary.

**Supplementary Information:**

The online version contains supplementary material available at 10.1186/s13104-022-06049-5.

## Introduction

The provincial and local governments across Canada have cooperated to impose measures to reduce the spread of SARS-CoV-2 impact on the economy. Implementing public health measures [1–3], such as physical distancing between individuals (at least six feet) and wearing masks, is considered an available and effective policy for preventing and reducing severe COVID-19 infection [4–6].

In Manitoba, a Pandemic Response System Called “RestartMB” was introduced to provide a clear and responsive overview of the current risk of COVID-19 and the measures taken. Accordingly, the Response System sets out a four colour-coded level system: critical (red), restricted (orange), caution (yellow), and limited risk (green) levels [7]. On February 17, 2020, the Chief Public Health Officer of Manitoba established an incident command structure to link public health with the acute health care system to make pandemic response planning easier and faster.

During the estimated first wave, starting March 12 (first reported case) to July 17, 2020 (lowest number of cases between waves), the numbers were relatively low compared to other Canadian provinces such as Ontario that reported 15,728 confirmed cases on April 28, 2020 [8–10]. However, this number increased drastically, reaching 34,174 positive cases on 31 March 2021 [7] and 131,047 by February 28, 2022 [11]. Currently, there is no timeline to demonstrate the effectiveness of the implemented mitigation measures in controlling the burden of COVID-19. The aim of this study is to describe the incidence of COVID-19 relative to the public health measures implemented in Manitoba using the COVID-19 dataset.

## Main text

### Methods

We used data from Manitoba Health and Senior Care (MHSC) to examine the temporal trends of daily reported COVID-19 cases and the coinciding public health measures implemented from March 12, 2020 to February 28, 2022. MHSC operates under the obligations of the Minister of Health and Seniors Care legislation and responsibilities and reports the number of daily confirmed COVID-19 cases [12].

Using the MHSC COVID-19 dataset, we conducted a descriptive population-based study. We compared the number of COVID-19 positive cases before, during, and after the implementation of mitigation measures during the first year (12 March, 2020–28 February, 2021) and second year (1 March, 2021–28 February, 2022) of the pandemic. The 7-day moving average and the crude COVID-19 infection rate per 100,000 Manitobans were calculated.

## Results

**Fig. 1:**
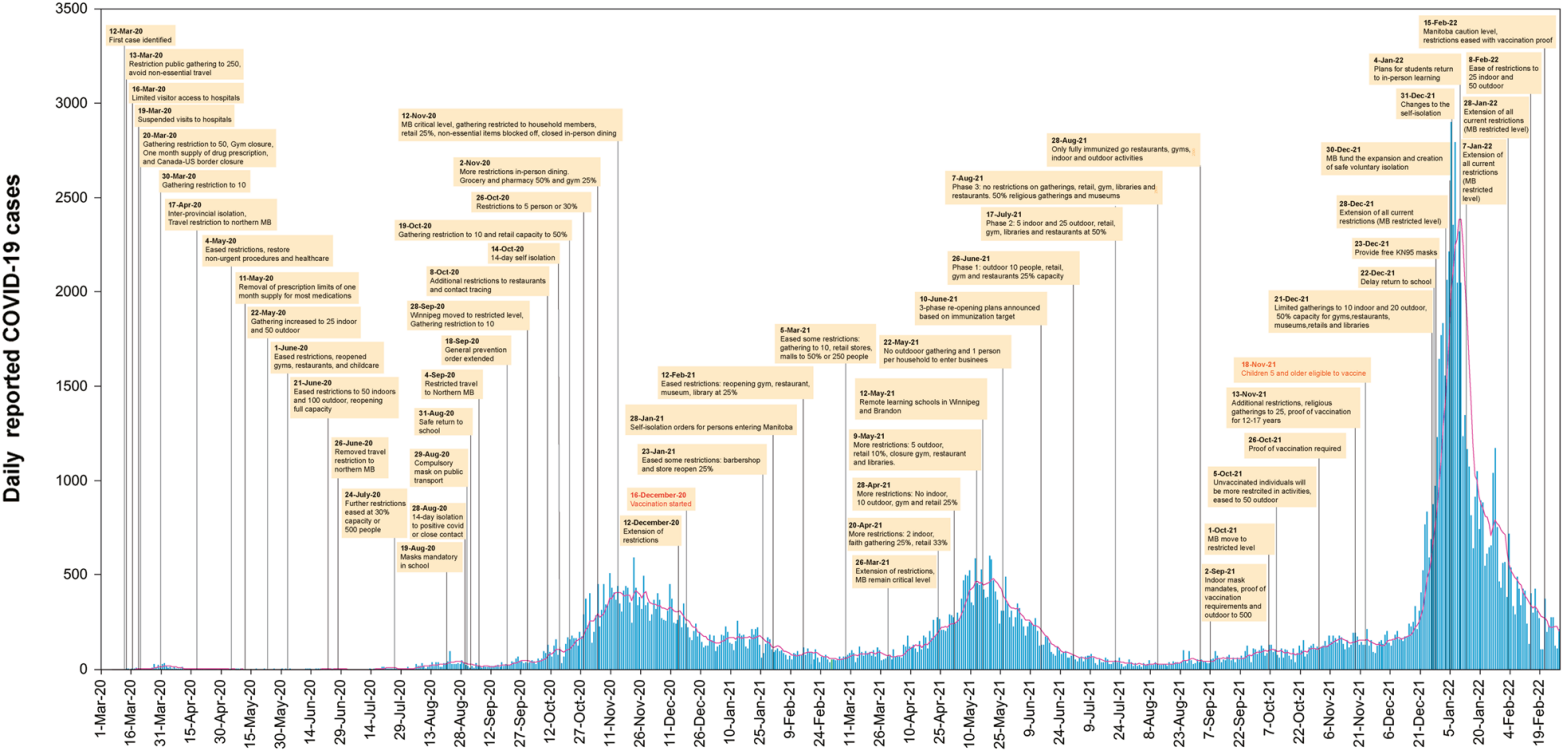
Measures applied and reported COVID-19 cases in Manitoba (MB) from March 1, 2020 to February 28, 2022. (Red line represents the 7-day moving average of reported cases)

In our study, we examined the entire Manitoba population of approximately 1.38 million Manitobans. Manitoba reported the first confirmed COVID-19 case on March 12, 2020 and the Premier of Manitoba declared a state of emergency for 30 days on March 20. From March 13 to March 31, 2020, several mitigation strategies were applied, including physical distancing, limiting the number of gatherings on March 13 to 250 people and then to 50 on March 20; and lastly to 10 people on March 30. Additional provincial orders were provided to businesses, communities, and health-care services including closure of universities and suspending classes for school’s kindergarten-12, non-urgent medical care restriction, limiting one-month supply of drug prescriptions, non-essential travel limitations, and closure of the Canada–US border to non-essential travel (Additional file 1: Table S1). On April 17, 2020, the government issued new public health orders, which prevented travel to Northern Manitoba and implemented a mandatory 14-days self-isolation for people entering Manitoba who travelled inter-provincially. Following the implementation of these restrictions, the infection rate/100,000 Manitobans decreased from 2.4 (April 1) to 0.07 infections (May 1, 2020) (Fig. 1).

Between May 4 and July 17, 2020, the reported cases stabilized, and some restrictions were lifted. During this phase, retail businesses, museums, libraries, gyms, barbers, restaurants, and bars were reopened and travel restriction to Northern Manitoba was removed. Additionally, gathering restrictions were eased; up to 100 people outdoor and up to 50 indoor were permitted, but physical distancing continued to be mandatory.

**Fig. 2:**
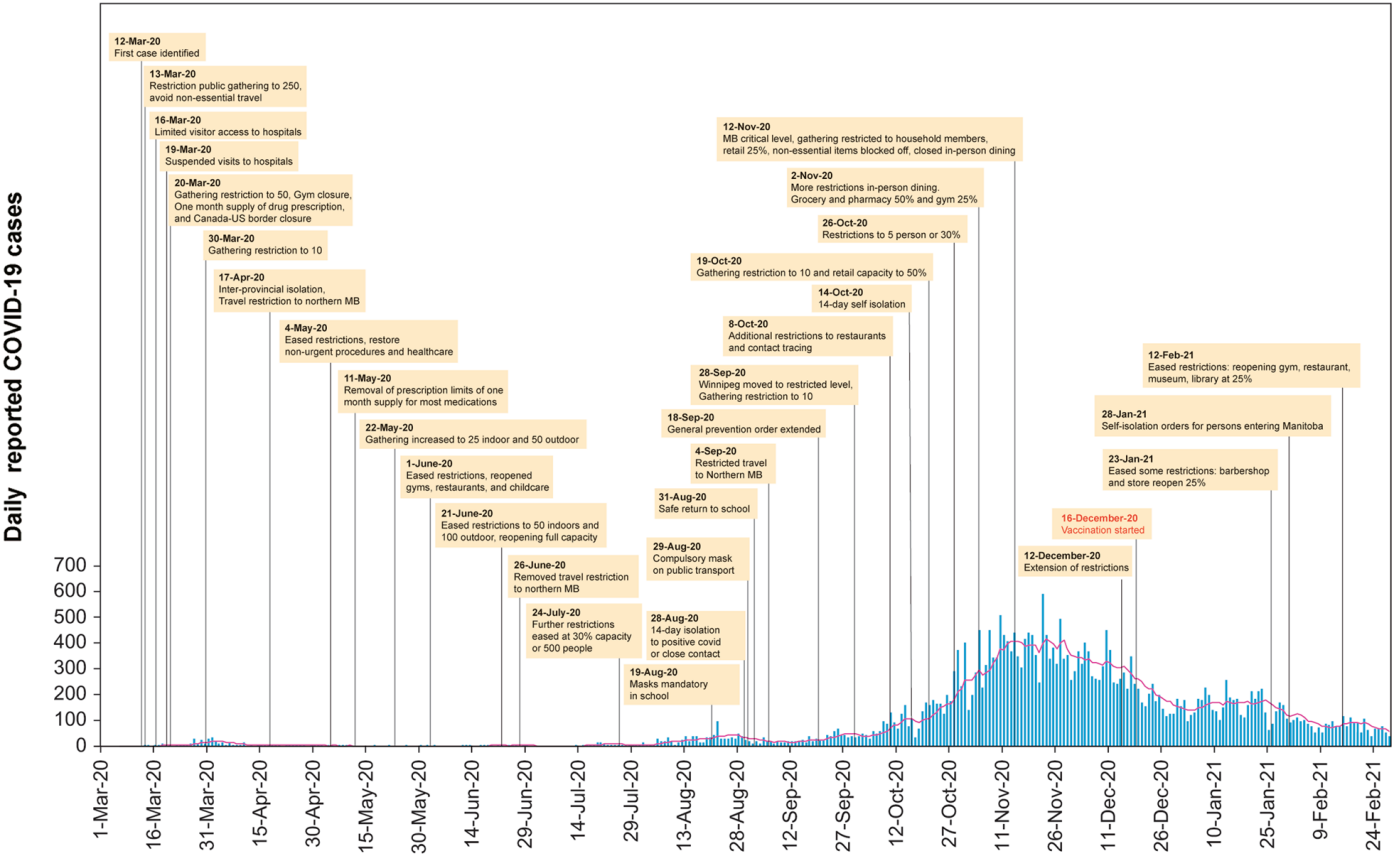
Measures applied and reported COVID-19 cases in Manitoba (MB) from March 1, 2020 to February 28, 2021. (Red line represents the 7-day moving average of reported cases)

As the beta variant of COVID-19 began emerging, the infection rate increased from 0.43 on July 17 to 1.1 infections/100,000 Manitobans on August 18, 2020. Additional restrictions were implemented, with compulsory 14-day self-isolation to those who tested positive or were exposed to COVID-19 through close contact on August 28 and mandatory use of masks on public transport as of August 29. Before August 29, the use of masks was not enforced. On August 31, public health officials and Manitoba Education revealed a plan to safely return children to school in Fall 2020 (Fig. 2).

The province extended the state of emergency for the sixth time on September 10. On September 28, Winnipeg was declared at the restricted level (one level below the highest), public gatherings were limited to 10 people and the use of masks was mandatory in public spaces. During September and October, there were several outbreaks in Personal Care Homes and Long-Term Care in Manitoba. In addition, as of October 30, the ICU capacity of hospitals has almost reached the full capacity of COVID-19. During the second wave, the infection rate increased from 4.2 on October 7 to 11.5 infections/100,000 Manitobans on October 14, 2020. Since October 19, Manitoba had three-digit daily cases for three consecutive months, with a peak achieved during November 4–27, 2020 (ranging from 227 to 593). (Fig. 2).

The surge in COVID-19 infection rate prompted the province to implement additional restrictions from October-January 2021. Public health orders limited social contacts to household members, non-essential items could not be purchased in-store, closure of gyms and restaurants, with all of Manitoba placed in the highest response level (“critical”) in November 2020. The daily reported cases stabilized during December 18 through January 29, 2021 (ranging from 64 to 257), and the infection rate consequently reduced from 7.5 (January 30) to 2.75 infections/ 100,000 Manitobans (February 28, 2021).

Manitoba announced a new phase on February 12 that eased some restrictions including a plan to reopen gyms, museums, libraries, malls and allowing reopening of restaurants at 25% capacity. However, physical distancing and use of masks were still mandatory. Updated guidance from the public health officials extended these measures while expanding the capacity to 50% and outdoor gatherings to 10 people on March 5, 2021. The COVID-19 daily reported case count remained relatively low ranging from 50 to 121 cases during March 2021.

**Fig. 3:**
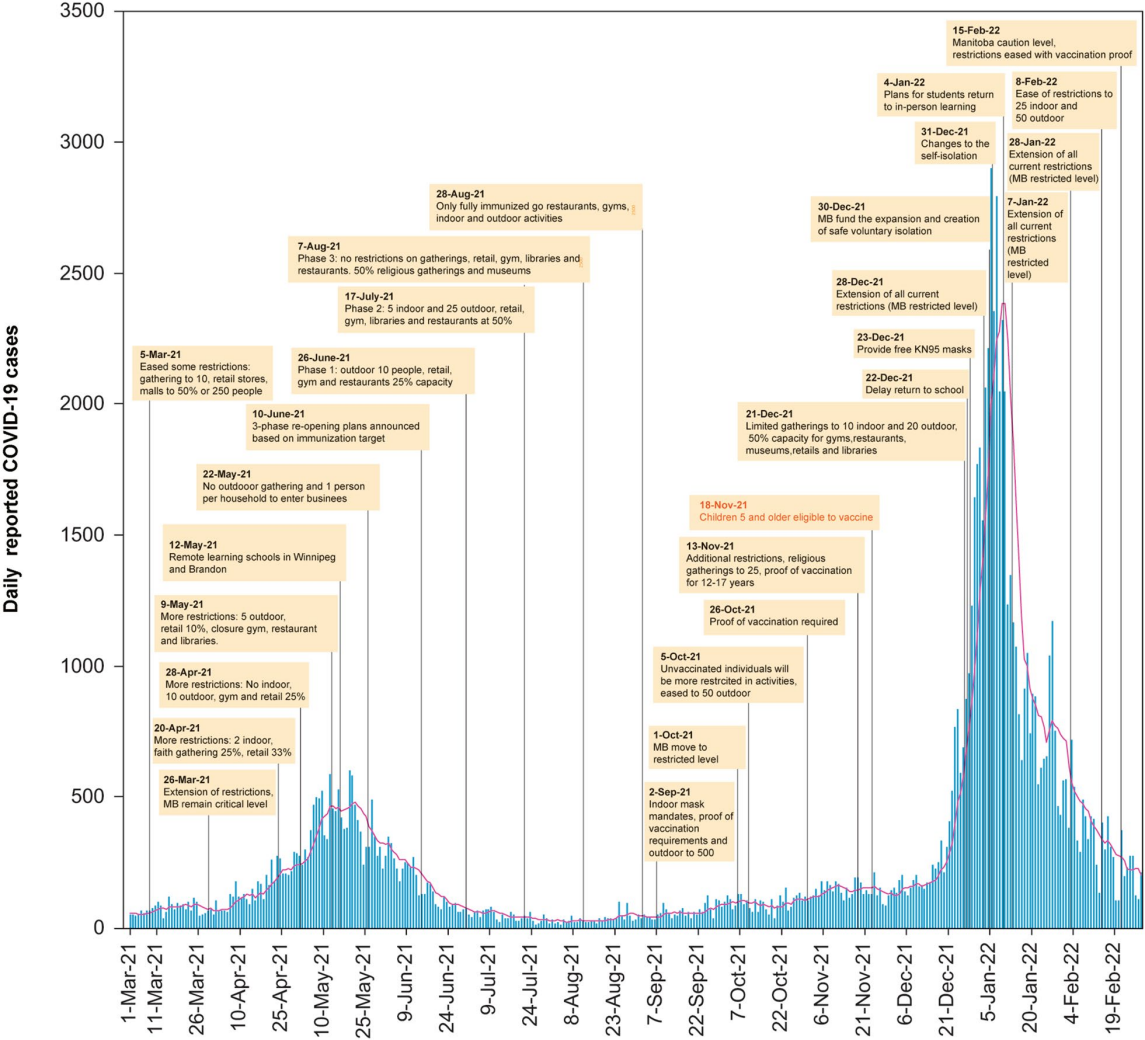
Measures applied and reported COVID-19 cases in Manitoba (MB) from March 1, 2021 to February 28, 2022. (Red line represents the 7-day moving average of reported cases)

During April and May 2021, the gamma variant was reported in Manitoba, and the infection rate peaked on May 12 at 42.5/100,000. The COVID-19 vaccination rates were low with only 5.85% of the eligible population fully vaccinated and 34.66% partially vaccinated, as of May 15. Further restrictions were implemented from April 20 to May 22, limiting gatherings to only household members and retail capacity to 10%. Additionally, as ICU numbers surged, Manitoba sent ICU patients to hospitals in other provinces, including Ontario and Saskatchewan (Fig. 3).

Between June 19 and September 17, 2021, the reported cases stabilized, some restrictions were eased, and Manitoba started the 3 phase re-opening plans on June 26. During the re-opening phase, the strategy was to lift public health orders based on meeting pre-set immunization targets (phase 1 both doses target is 25% and 75% for phase 3). Among Manitobans 12 years and older, 31.46% received both doses on June 26 and increased to 62.77% on August 7. Accordingly, the province entered phase three of the re-opening plan that lifted restrictions on gatherings, and visiting restaurants, retail capacity, gyms, and libraries. To boost vaccination rates among unvaccinated Manitobans, proof of double vaccination was required for provincial employees, restaurants, gyms, indoor activities, and sport events as of August 28, 2021.

By October 1, more than 70% of eligible Manitobans were fully vaccinated, but around 400,000 individuals were unvaccinated, and the number of Delta variant cases and hospitalizations increased. The province moved into “restricted” level and new public health orders were announced that restricted multiple activities to those who were unvaccinated residents but were eligible to be vaccinated.

The infection rate per 100,00 Manitobans increased from 8.6 (November 1) to 128 (December 31), and additional prevention strategies were implemented. On November 18, 2021, children aged five years and older were eligible for the pediatric Pfizer-BioNTech vaccine. The province reported its first Omicron variant on December 7, with peak omicron cases reached on January 7, 2022 (202.2 infections/100,00). Mitigation measures were extended through January and by January 28, 38.31% were vaccinated with a booster dose while 82.55% received at least one dose of vaccine. Following the extension of the restrictions, the infection rate dropped to 21.8/100,000 on February 15, 2022, and Manitoba moved to the second lowest restriction level (“caution”), however, proof of vaccination was still required for most activities (Fig. 3).

## Discussion

During the first wave, the daily number of COVID-19 cases in Manitoba declined after the widespread mitigation measures including physical distancing, gathering restrictions, closure of several facilities and businesses. Whereas during the second wave—upon lifting some of the restrictions—numbers increased exponentially, which required more strict strategies, such as mandated mask wearing, limiting visits to household members, closure of many public commercial spaces. All of Manitoba was declared to be at the highest Pandemic Response System level (“critical”). The number of positive cases stabilized during December and continued to decline into January. While some of the restrictions were lifted during January and February 2021, strict measures were re-introduced during the third wave of COVID-19 (April 2021). The healthcare system in Manitoba was distressed due to various COVID-19 variants and low vaccination rates. By August 2021, the province reached vaccination targets and eased some of the restrictions. However, in December 2021, the cases surged due to the omicron variant and restrictions were reintroduced. More than 38% of Manitobans were vaccinated with a third vaccine dose by February 2022.

In May 2020, World Health Organization recommended governments that before reopening, the positivity rate should be below 5% for at least 14 days [13]. As of November 25, 2020, the total number of tests exceeded 230,000 and the Manitoba five-day test positivity rate was higher than 14%, leading to more restrictive measures. However, by March 2021, as the total number of tests in Manitoba exceeded 590,000 and the positivity rate was below 5% province-wide, some restrictions were eased [7]. Changes in positivity rates were pivotal in driving government decisions in Manitoba, especially throughout the second wave.

Studies showed that vaccines were effective in reducing COVID-19 infection adverse outcomes, decreasing the rate of hospitalizations and ICU admission [14–16]. Several restrictive measures can effectively slow the spread of SARS-CoV-2 but the greatest effect is likely obtained by applying multiple measures [5, 17–19]. There is limited research on the impact of the public policies on COVID-19 incidence at provincial/state levels [5, 17, 20]. A report of COVID-19 incidence in Arizona showed that the mitigation measures including physical distancing, wearing masks and stay-at-home orders were associated with reducing community spread of SARS-CoV-2 by 75% [21].

### Limitations

Limitations of this report should be acknowledged. First, the extent to which positive SARS-CoV-2 cases will increase once the mitigations strategies are relaxed depends on several difficult-to-measure factors, such as the individuals’ adherence to those measures (e.g., limitation of gatherings and mask-wearing). Second, due to differences in mitigation strategies and population composition in Manitoba, Canada, findings might not be generalizable. Moreover, our study is an ecological study so we cannot correlate the decrease in COVID-19 cases with the measures applied in a robust causal text. Third, PCR or molecular testing was restricted to symptomatic nurses and other vulnerable populations as of March 26, 2020 and became available to all Manitobans only by April 28, 2020. Fourth, as the data only represent reported cases, there are underreported cases among non-tested individuals and indigenous populations. It should also be acknowledged that a delay between policy change and the population effects on COVID-19 cases is expected given the delay in developing symptoms and getting tested. Additionally, we did not estimate the lag effect of the mitigation measures.

This report provides evidence for the effectiveness of public health measures in reducing SARS-CoV-2 cases. Physical distancing, gathering restrictions and wearing masks are instrumental in preventing the spread of the virus. Also, we anticipate it can help policy makers reinforce effective measures that allow enhanced prevention, awareness, and response to future outbreaks and pandemics. Future studies into the extent of the implementation and adherence of the public health restrictions are necessary.

## Supplementary Information

**Additional file 1: Table S1:** Daily reported cases, average 7-day infection rate and public health measures applied in Manitoba, Canada from March 2020 up to February 2022.

## Data Availability

The datasets generated and/or analysed during the current study are available in the Manitoba Health and Senior Care COVID-19 repository, [https://www.gov.mb.ca/covid19/updates/cases.html#maps].

## Abbreviations

COVID-19: Coronavirus 2019
MHSC: Manitoba Health and Seniors Care
SARS-CoV-2: Severe acute respiratory syndrome Coronavirus-2
ICU: Intensive Care Unit
