# Supplementary material for: Trends of COVID-19 incidence in Manitoba and public health measures: March 2020 to February 2022
Source: BMC Res Notes. 2022 May 10;15:162. doi: 10.1186/s13104-022-06049-5 (PMC9088149; doi:10.1186/s13104-022-06049-5)
Supplement: Supplementary file 1 — Additional file 1: Table S1: Daily reported cases, average 7-day infection rate and public health measures applied in Manitoba, Canada from March 2020 up to February 2022. [file 13104_2022_6049_MOESM1_ESM.docx]

**Table S1:** Daily reported cases, average 7-day infection rate and public health measures applied in Manitoba, Canada from March 2020 up to February 2022.

| **Date** | **Daily reported cases** | **Rate/100,000**  **(Average 7-day)** | **Measures applied** |
| --- | --- | --- | --- |
| **13-Mar-20** | 2 | 0.02 | Restriction public gathering to 250, avoid non-essential travel |
| **16-Mar-20** | 3 | 0.06 | Limited visitor access to hospitals |
| **19-Mar-20** | 1 | 0.16 | Suspended visits to hospitals |
| **20-Mar-20** | 1 | 0.15 | Gathering restriction to 50, Gym closure, One month supply of drug prescription, and Canada-US border closure. |
| **30-Mar-20** | 9 | 0.88 | Gathering restriction to 10 |
| **17-Apr-20** | 5 | 0.16 | Inter-provincial isolation, Travel restriction to northern MB |
| **04-May-20** | 1 | 0.08 | Eased restrictions, restore non-urgent procedures and healthcare |
| **11-May-20** | 0 | 0.08 | Removal of prescription limits of one month supply for most medications |
| **22-May-20** | 0 | 0.03 | Gathering increased to 25 indoor and 50 outdoor |
| **01-Jun-20** | 3 | 0.06 | Eased restrictions, reopened gyms, restaurants, and childcare |
| **21-Jun-20** | 1 | 0.07 | Eased restrictions to 50 indoors and 100 outdoor, reopening full capacity |
| **26-Jun-20** | 4 | 0.29 | Removed travel restriction to northern MB |
| **24-Jul-20** | 6 | 0.56 | Further restrictions eased at 30% capacity or 500 people |
| **19-Aug-20** | 33 | 2.01 | Masks mandatory in school |
| **28-Aug-20** | 47 | 2.97 | 14-day isolation to positive covid or close contact |
| **29-Aug-20** | 36 | 2.33 | Compulsory mask on public transport |
| **31-Aug-20** | 18 | 2.20 | Safe return to school |
| **04-Sep-20** | 33 | 1.53 | Restricted travel to Northern MB |
| **18-Sep-20** | 17 | 1.52 | General prevention order extended |
| **28-Sep-20** | 35 | 3.39 | Winnipeg moved to restricted level, Gathering restriction to 10 |
| **08-Oct-20** | 97 | 3.86 | Additional restrictions to restaurants and contact tracing |
| **14-Oct-20** | 160 | 7.84 | 14-day self isolation |
| **19-Oct-20** | 136 | 7.33 | Gathering restriction to 10 and retail capacity to 50% |
| **26-Oct-20** | 200 | 12.01 | Restrictions to 5 person or 30% |
| **02-Nov-20** | 197 | 18.71 | More restrictions in-person dining. Grocery and pharmacy 50% and gym 25% |
| **12-Nov-20** | 409 | 29.01 | MB critical level, gathering restricted to household members, retail 25%, non-essential items blocked off, closed in-person dining. |
| **12-Dec-20** | 246 | 22.42 | Extension of restrictions |
| **16-Dec-20** | 224 | 21.54 | Vaccination started |
| **23-Jan-21** | 224 | 12.71 | Eased some restrictions: barbershop and store reopen 25% |
| **28-Jan-21** | 167 | 10.55 | Self-isolation orders for persons entering Manitoba |
| **12-Feb-21** | 97 | 5.37 | Eased restrictions: reopening gym, restaurant, museum, library at 25% |
| **05-Mar-21** | 66 | 3.76 | Eased some restrictions: gathering to 10, retail stores, malls to 50% or 250 people. |
| **26-Mar-21** | 49 | 6.20 | Extension of restrictions, MB remain critical level |
| **20-Apr-21** | 162 | 11.17 | More restrictions: 2 indoor, faith gathering 25%, retail 33% |
| **28-Apr-21** | 219 | 16.10 | More restrictions: No indoor,10 outdoor, gym and retail 25% |
| **09-May-21** | 522 | 30.35 | More restrictions: 5 outdoor, retail 10%, closure gym, restaurant and libraries. |
| **12-May-21** | 586 | 33.83 | Remote learning schools in Winnipeg and Brandon |
| **22-May-21** | 413 | 33.68 | No outdoor gathering and 1 person per household to enter business |
| **10-Jun-21** | 230 | 16.83 | 3-phase re-opening plans announced based on immunization target |
| **26-Jun-21** | 96 | 6.69 | Phase 1: outdoor 10 people, retail, gym and restaurants 25% capacity |
| **17-Jul-21** | 50 | 3.08 | Phase 2: 5 indoor and 25 outdoor, retail, gym, libraries and restaurants at 50% |
| **07-Aug-21** | 46 | 1.95 | Phase 3: no restrictions on gatherings, retail, gym, libraries and restaurants. 50% religious gatherings and museums |
| **28-Aug-21** | 48 | 4.06 | Only fully immunized go restaurants, gyms, indoor and outdoor activities |
| **02-Sep-21** | 50 | 3.53 | Indoor mask mandates, proof of vaccination requirements and outdoor to 500 |
| **01-Oct-21** | 103 | 6.63 | MB move to restricted level |
| **05-Oct-21** | 84 | 7.07 | Unvaccinated individuals will be more restricted in activities, eased to 50 outdoor |
| **26-Oct-21** | 116 | 7.54 | Proof of vaccination required |
| **13-Nov-21** | 105 | 11.06 | Additional restrictions, religious gatherings to 25, proof of vaccination for 12-17 years |
| **18-Nov-21** | 194 | 10.63 | Children 5 and older eligible to vaccine |
| **21-Dec-21** | 409 | 20.57 | Limited gatherings to 10 indoor and 20 outdoor, 50% capacity for gyms, restaurants, museums, retails and libraries. |
| **22-Dec-21** | 523 | 23.46 | Delay returns to school |
| **23-Dec-21** | 769 | 29.08 | Provide free KN95 masks |
| **28-Dec-21** | 972 | 54.43 | Extension of all current restrictions (MB restricted level) |
| **30-Dec-21** | 1646 | 70.84 | MB fund the expansion and creation of safe voluntary isolation |
| **31-Dec-21** | 1770 | 80.52 | Changes to the self-isolation |
| **04-Jan-22** | 2212 | 127.40 | Plans for students return to in-person learning |
| **07-Jan-22** | 2791 | 162.61 | Extension of all current restrictions (MB restricted level) |
| **28-Jan-22** | 752 | 56.15 | Extension of all current restrictions (MB restricted level) |
| **08-Feb-22** | 427 | 32.97 | Ease of restrictions to 25 indoor and 50 outdoor |
| **15-Feb-22** | 301 | 23.42 | Manitoba caution level, restrictions eased with vaccination proof |

MB: Manitoba; Rate of infection per 100,000 = (number of 7-day average reported cases/ Manitoba population [1380,000])*100,000.
